# Determinants of Clinician Knowledge on Aging and HIV/AIDS: A Survey of Practitioners and Policy Makers in Kampala District, Uganda

**DOI:** 10.1371/journal.pone.0057028

**Published:** 2013-02-28

**Authors:** Ekwaro A. Obuku, Sujal M. Parikh, Victoria Nankabirwa, Nelson I. Kakande, David K. Mafigiri, Harriet Mayanja-Kizza, Cissy M. Kityo, Peter N. Mugyenyi, Robert A. Salata

**Affiliations:** 1 Joint Clinical Research Centre, Kampala, Uganda; 2 Department of Medicine, Division of Infectious Diseases and HIV Medicine, Case Western Reserve University, Cleveland, Ohio, United States of America; 3 University of Michigan Medical School, Ann Arbor, Michigan, United States of America; 4 Department of Epidemiology, Mailman School of Public Health, Columbia University, New York, New York, United States of America; 5 Department of Social Work and Social Administration, College of Humanities and Social Sciences, Makerere University, Kampala, Uganda; 6 Department of Medicine, College of Health Sciences, Makerere University, Kampala, Uganda; 7 Center for Social Science Research on AIDS, Department of Anthropology, College of Arts and Sciences, Case Western Reserve University, Cleveland, Ohio, United States of America; University of Pittsburgh Medical Center, United States of America

## Abstract

**Objective:**

The HIV/AIDS epidemic has evolved with an increasing burden in older adults. We assessed for knowledge about aging and HIV/AIDS, among clinicians in Kampala district, Uganda.

**Methods:**

A cross-sectional survey of 301 clinicians complemented by 9 key-informant interviews between May and October 2011. Data was analyzed by multivariable logistic regression for potential determinants of clinician knowledge about HIV/AIDS in older adults, estimating their adjusted Odds Ratios (aOR) and 95% confidence intervals (95% CI) using Stata 11.2 software.

**Results:**

Two-hundred and sixty-two questionnaires (87.7%) were returned. Respondents had a median age of 30 years (IQR 27–34) and 57.8% were general medical doctors. The mean knowledge score was 49% (range 8.8%–79.4%). Questions related to co-morbidities in HIV/AIDS (non-AIDS related cancers and systemic diseases) and chronic antiretroviral treatment toxicities (metabolic disorders) accounted for significantly lower scores (mean, 41.7%, 95% CI: 39.3%–44%) compared to HIV/AIDS epidemiology and prevention (mean, 65.7%, 95% CI: 63.7%–67.7%). Determinants of clinician knowledge in the multivariable analysis included (category, aOR, 95% CI): clinician age (30–39 years; 3.28∶1.65–9.75), number of persons with HIV/AIDS seen in the past year (less than 50; 0.34∶0.14–0.86) and clinical profession (clinical nurse practitioner; 0.31∶0.11–0.83). Having diploma level education had a marginal association with lower knowledge about HIV and aging (p = 0.09).

**Conclusion:**

Our study identified gaps and determinants of knowledge about HIV/AIDS in older adults among clinicians in Kampala district, Uganda. Clinicians in low and middle income countries could benefit from targeted training in chronic care for older adults with HIV/AIDS and long-term complications of antiretroviral treatment.

## Introduction

HIV/AIDS is a major public health problem globally, which disproportionately affects sub-Saharan Africa [1]. The first case of HIV/AIDS in Uganda was reported in early 1980s, at Kasensero landing site, Rakai [2] which was about the time America documented its index case in Los Angeles [3]. Since then several advancements in HIV/AIDS prevention, care, treatment and laboratory monitoring have prolonged the survival of persons with HIV/AIDS [4]–[10].

The world population is aging [11], even among people living with HIV/AIDS (PLWHAs) [12], [13]. The HIV/AIDS epidemic has evolved resulting in a rising burden of older adults who are 50 years and older [14], and is characterized by new serious non-AIDS related events including cardiac, renal, liver manifestations as well as non-AIDS cancers [12], [15], [16]. These events have been observed at first presentation with HIV or among those who were younger at initial HIV diagnosis and survived following successful treatment with antiretroviral-therapy. At the same time, non-communicable diseases are on the rise in sub-Saharan Africa [17] and their interaction with existing infectious diseases especially HIV/AIDS is still unclear but certainly important.

Although evidence on aging and HIV/AIDS is largely from America and Europe, Low– and Middle Income Countries (LMICs) are beginning to observe this phenomenon. Studies from Uganda showed that age at HIV sero-conversion was associated with more rapid progression to AIDS [18] and younger patients (18–49 years) had better survival than older patients (≥50 years) even after initiation of antiretroviral therapy [19]. In Uganda general medical doctors, clinical officers, clinical nurses and specialist physicians attend to PLWHAs. It is not known how the local health systems especially clinicians have responded to this inevitable dual challenge of aging in HIV/AIDS and non-communicable diseases.

Health care worker knowledge on HIV/AIDS has been shown to correlate with attitudes, practices and outcomes [20], [21], [22]. Thus, there is a need to assess clinician knowledge about managing older adults with HIV/AIDS especially in LMICs. From an extensive literature search, few studies have examined clinician knowledge on aging and HIV [23], [24], [25], [26], and these were in the USA. Our study objective was to investigate clinician knowledge about aging in persons with HIV/AIDS - in Kampala district Uganda; in order to identify training needs and inform healthcare decision making.

## Methods

### Study Design, Procedures and Sample Size Estimation

This was a mixed methods study conducted between May and October 2011 [27]. The first part employed a cross-sectional survey of clinicians using self – administered structured questionnaires. We defined a clinician as a medical doctor or clinical officer actively participating in patient care. In Uganda, clinical officers would have completed a shorter medical diploma training of three years, compared to the five years degree course by general medical doctors. Notably, in Kampala HIV care clinics medical doctors provide first – line clinical care to patients with acute illnesses, whilst clinical officers and nurses majorly cater for stable patients. Respondents were consecutively enrolled from all the major health care facilities in Kampala district including: public (n = 1), private–for –profit (n = 5) and private–not–for–profit hospitals (n = 2); health centres (n = 3) and free–for–service HIV/AIDS care centres of excellence (n = 8) [28]. Nurses were included if they worked in HIV/AIDS wards, chronic care or research clinics. Surgeons, pediatricians or nurses working in surgery or pediatrics were excluded.

Since the expected level of aging and HIV/AIDS related knowledge among clinicians in Uganda was unknown, a maximum variability of 0.5 was assumed adjusting for a small population, N = 500, and 0.1 proportion of incomplete or non-response; 0.05 precision and 1.96 standard normal deviate. The total required sample size was estimated at 239 clinicians using Cochran’s formula [29].

The second method constituted 9 key informants who were purposively sampled and interviewed. Snowball sampling was used to further recruit more respondents until no new information was obtained. These included: senior clinicians working in HIV/AIDS clinics (n = 3); HIV/AIDS activists (n = 3); and policy makers in the relevant government institutions (n = 3).

### Survey Questionnaire and Key Informant Guide

The study questionnaire adopted aspects of instruments previously used for similar evaluations [21], [22], [23], [25] with modifications to suit investigations about aging and HIV/AIDS in Uganda. This questionnaire was piloted and had a Cronbach’s alpha coefficient of 0.68 for 34 knowledge items [30]. The key informant guide constituted open-ended questions and was administered orally. The knowledge questionnaire has been included here–in as an additional file (Figure S1).

### Exposure Variables

These included: clinician age, gender, education level, professional cadre, work setting, interest in geriatrics, history of taking an HIV test, years of working experience and additional training in HIV/AIDS.

### Outcome Measures

Clinician knowledge about aging and HIV/AIDS was the main outcome measure. This examined HIV/AIDS epidemiology and prevention, immunology, antiretroviral treatment outcomes and laboratory results, non-communicable co-morbidities and long term complications of antiretroviral treatment scored as “True, False or Don’t Know”. Responses to the 34-questions on knowledge about aging and HIV/AIDS were scored (one point for correct and zero for incorrect), then aggregated into those who scored 50% or below, and those above 50%. The key informant guide captured respondents’ knowledge, experiences and recommendations about prevention, care and treatment of HIV/AIDS among older adults in Uganda.

### Statistical Considerations

Data was coded, entered in Epi Info™ version 3.5.3 (Centres for Disease Control and Prevention, Atlanta, USA) and analyzed using Stata™ version 11.2 (StataCorp, College Station, Texas, USA). This data was summarized using frequencies, percentages and appropriate measures of central tendency. The association between exposure variables and knowledge score was explored using Student’s t-test, one-way analysis of variance and univariate logistic regression. Tests were two-sided and significant if *P*<0.05. Potential predictors with *P*<0.1 at univariate level were further explored in a multivariable logistic regression model to estimate the adjusted Odds Ratios and their 95% confidence intervals. Multiple imputations by chained equations was employed for sensitivity analysis of the missing data using the Stata™ command “ice” and “mim”, assuming a Missing At Random mechanism. In the adjusted analysis, all missing baseline variables and the outcome, HIV/AIDS knowledge, were included to create twenty imputed data sets. Qualitative data from key informant interviews were transcribed and analyzed for thematic content on clinician knowledge and training, then triangulated with the survey findings.

### Ethical Considerations

This study was approved by the Joint Clinical Research Centre Institutional Review Board and Uganda National Council for Science and Technology (HS 990). Clinicians were given a written informed consent guide and asked to tick yes or no before completing the anonymised survey.

## Results

### Clinician Background and Practice Characteristics

Three hundred and one questionnaires were distributed of which 262 (87.7%) were returned. Clinicians who completed the survey had a median age of 30 years (IQR: 27–34). The majority of respondents (59.7%) were male and 57.8% were general medical doctors. Most respondents worked in a hospital setting (69.7%) and the median time of clinical experience was 4 years (IQR: 2–7). Seventy two percent qualified with a bachelor’s degree and 88.8% had received additional short-term training in HIV/AIDS covering the following topics: prevention of mother-to-child transmission (45.8%), TB/HIV co-infection (44.3%), antiretroviral-therapy (40.8%) whilst only 23.6% completed modules in HIV/AIDS co-morbidities which predominantly cover non-communicable diseases and complications of chronic antiretroviral-therapy. At least 90% of the respondents had been tested for HIV before and 87% said they had interest in geriatrics whilst about a half (52.4%) had attended to over 100 PHLWAs in the preceding year (Table 1).

**Table 1:** Background Characteristics and HIVAIDS Knowledge Scores for Clinicians in Kampala District.

Category	N = 262 (%)	Mean Score	95% CI
**Age**			
20–29	120 (47.2)	46.9	44.8, 49.1
30–39	97 (38.2)	*55.2	53.0, 57.5
>40	37 (14.6)	46.5	41.8, 51.2
**Gender**			
Male	151 (59.7)	51.8	49.9, 53.7
Female	102 (40.3)	*46.9	44.2, 49.7
**Professional Cadre**			
Medical Officer	144 (57.8)	50.8	48.7, 52.8
Nurse	38 (15.3)	*44.6	40.2, 48.9
Clinical Officer	29 (11.7)	48.9	44.3, 53.6
Specialist Physician	38 (14.3)	53.3	49.1, 57.5
**Work Setting**			
Hospital Ward	168 (69.7)	49.2	47.3, 51.2
HIV/AIDS Clinic	53 (22)	52.7	49.6, 55.9
Health Centre	20 (8.3)	50.3	45.1, 55.5
**Clinical Experience (Years)**			
<3	106 (44.5)	47.1	44.8, 49.3
4–6	63 (26.5)	*52.6	49.4, 55.8
7–9	34 (14.3)	*54.4	50.2, 58.7
>9	35 (14.7)	50.6	45.8, 55.4
**Highest Education**			
Degree	179 (72.5)	49.7	47.8, 51.6
Diploma	28 (11.3)	45.8	41.3, 50.3
Masters	40 (16.2)	53.7	49.5, 57.9
**Additional HIV Trainings**			
Yes	231 (88.8)	50.5	48.8, 52.1
No	29 (11.2)	*44	39.3, 48.8
**Topic of Short Training**			
PMTCT	120 (45.8)	51.0	48.6, 53.5
TB/HIV co-infection	116 (44.3)	50.6	48.2, 52.9
Antiretroviral Therapy	107 (40.8)	50.8	48.3, 53.2
HIV Counseling & Testing	97 (36.6)	50.4	47.7, 53.1
HIV/AIDS Conferences	79 (29.8)	φ52.6	50.3, 54.9
Pediatrics & Adolescent HIV	69 (26)	48.7	45.6, 51.8
NCD Co-morbidities	62 (23.6)	φ53.7	50.6, 56.7
Sexual & Reproductive Health	55 (20.9)	50.9	47.4, 54.5
**Interest in Geriatrics**			
Yes	226 (86.9)	49.2	47.5, 50.9
No	34 (13.1)	53.4	49.1, 57.6
**Ever taken HIV/AIDS test**			
Yes	234 (90)	49.5	47.9, 51.2
No	26 (10)	51.5	45.8, 57.4
**PHAs seen in past year**			
>100	132 (52.4)	53.9	51.9, 55.9
50–100	58 (23)	*47.9	44.8, 51.2
<50	62 (24.6)	*44.6	41.6, 47.7
**PHAs >50 years seen**			
<25%	137 (62.3)	50.6	48.5, 52.8
>25%	83 (37.7)	51.9	49.2, 54.6

* Significant difference (p<0.05) compared to the first category;

φ compared to those without specific training Abbreviations: HIV – Human Immunodeficiency Virus; NCD – Non Communicable Diseases; PMTCT – Prevention of Mother to Child Transmission; PHAs – Persons Living with HIV/AIDS; TB – Tuberculosis; CI – Confidence Interval.

### Clinician Knowledge of Aging and HIV/AIDS

The mean knowledge score was 49% (range 8.8%–79.4%). Table 2 shows that questions about HIV/AIDS co-morbidities and chronic antiretroviral-therapy toxicities accounted for a lower mean score of 41.7% (95% CI: 39.3–44) compared to epidemiology and prevention which had the highest mean score, 65.7% (95% CI: 63.7–67.7). This low score was not significantly different from that of management of antiretroviral-therapy in persons with AIDS. Specific areas of extremely low scores included: understanding the immune-pathology of HIV in older adults in terms of chronic immune activation (12.2%), inflammation (11.5%) and myocardial infarction (16.4%); non-communicable co-morbidities manifesting as serious non-AIDS related events (11.3%); AIDS wasting syndrome (12.5%); distinguishing HIV prevalence by age strata (23.3%); appreciating the possibility of both hetero- and homosexual transmission among older adults (36.3%); ranking the interaction between TB and HIV or diabetes or tobacco smoking (18.7%) and the effectiveness of antiretroviral-therapy in prolonging life expectancy compared to the HIV negative population (11.8%).

**Table 2:** Clinician knowledge about HIVAIDS in older adults by thematic area (N = 262).

Knowledge area	*Correct % (95%CI)
**HIV Epidemiology and Prevention**	**65.7 (63.7–67.7)** §
Prevalence of HIV/AIDS in Uganda	74.1
Prevalence of HIV/AIDS among older adults (50–59 yrs) higher than younger adults (15–24 yrs)	23.3^φ^
HIV/AIDS transmission routes in older adults (including homo- or heterosexual practices)	36.3^φ^
Hetero-sexual intercourse not crucial risk factor for HIV transmission among older adults	80.5
Older adults without HIV/AIDS symptoms can be infectious	79.9
All pregnant women infected with HIV will have babies born with AIDS	86.6
Gloves are not necessary when handling body fluids from older adults	93.5
ARVs reduce the risk of Mother To Child Transmission to nearly 0%	51.2
**HIV Immunology**	**51.5 (49.4–53.6)** §
Older adults with negative result for HIV antibodies may be infected with the HIV/AIDS germ	68.3
HIV/AIDS is characterized by a decrease in CD4+ T-lymphocyte cells in the blood	84.7
An older adult with antibodies to the HIV virus is protected against HIV/AIDS	84.4
HIV/AIDS is characterized by chronic immune activation but not chronic inflammation	46.2
Features of chronic immune activation in the context of HIV	12.2^φ^
Features of chronic inflammation in the context of HIV	11.5^φ^
Myocardial Infarction in an older adult with HIV/AIDS is an event of the immune system	16.4^φ^
HIV/AIDS causes earlier ageing of the body associated with premature clinical outcomes	69.6
Older patients, at the time of initial HIV diagnosis, tend to have higher CD4 cells than younger individuals	70.3
**Antiretroviral Treatment**	**40.3 (37.6–42.9)** §
Older adults are less likely to use condoms, more likely to have multiple partners and those who are HIV infected adhere less to ART than younger individuals	50.8
Effective ART isn’t a cure but can improve average life expectancy of HIV infected older adults to as good as that of the HIV negative general population	11.8^φ^
The life expectancy of PHAs taking effective ART is similar to the general population only if CD4+ counts are ≥500 cells/mm^3^ for ≥5 years	46.8
Even after 5 years of effective ARVs, many patients fail to obtain normal CD4+ T-cell counts	56.3
Patients with CD4+ T-cell counts <500 cells/mm^3^ even after about 5 years of ARV use are unlikely to achieve ≥500 cells/mm^3^ during long-term follow-up	43.1
Older adults with HIV respond less well to ARVs compared to younger adults	41.8
The CD4+ cells peak response and plateau of older adults on the same ART regimen is lower than that of younger individuals	52.7
Older adults are likely to have undetectable viral loads more than younger individuals on ART	19.1^φ^
**Co-morbidities in HIV/AIDS and long term ART toxicities**	**41.7 (39.3–44)** §
Older patients with HIV have a shorter survival after a diagnosis of AIDS compared to younger patients	50.6
Conditions that constitute AIDS wasting syndrome	12.6^φ^
Rank the risk factors for suffering TB disease in older adults (HIV/AIDS, Diabetes, Smoking)	18.7^φ^
Serious Non-AIDS Related Events (SNARES) occur more frequently in older adults with HIV/AIDS even in the presence of effective ART use than in HIV negative older adults	11.5^φ^
HIV positive older adults have the same risk of co-morbidities (diabetes mellitus, non-AIDS related cancers, cardiovascular disease) as HIV negative older adults	49.4
Untreated HIV infection in older adults leads to protein energy malnutrition	71.5
ART causes initial loss of both central and limb fat (lipoatrophy) in older adults	45.8
After a period of time, ART causes gain of limb fat and central abdominal fat in older adults	43.1
Long term use of ART may cause redistribution of body fat increasing risk of DM in older adults	71.8

* Correct response may be **T**-True or **F**-False.

§ Mean score.

Φ Areas with extremely low scores (less than 40%).

### Determinants of Clinician Knowledge about Aging and HIV/AIDS

In the univariate analysis (Table 3) clinicians were more knowledgeable if they were between 30–39 years of age (OR = 4.56, 95% CI; 2.56–8.08); worked in a HIV/AIDS clinic (OR = 2.36, 95% CI; 1.25–4.47); had 3–6 or 7–9 years of clinical experience (OR = 2.54, 95% CI; 1.34–4.81; OR = 3.28, 95% CI; 1.47–7.30 respectively); had attained a masters degree (OR = 2.41, 95% CI; 1.18–4.91); or attended additional HIV training on co-morbidities (OR = 2.08, 95% CI; 1.16–3.70).

**Table 3:** Regression model for potential determinants of clinician Knowledge Scores.

Predictor Variable	Knowledge Score (%)		Unadjusted		Adjusted (N = 197)		Imputed (N = 262)	
	≤50	>50	OR	95% CI	aOR	95% CI	aOR	95% CI
**All Clinicians**	146 (55.7)	116 (44.3)						
**Age**								
20–29	83 (69.2)	37 (30.8)	1	**§**		**φ**		**§**
30–39	32 (36.1)	65 (63.9)	4.56	2.56–8.08	3.28	1.65–9.75	3.10	1.42–6.77
>40	27 (73)	11 (27)	0.95	0.42–2.10	0.46	0.08–1.78	0.48	0.13–1.79
**Gender**								
Male	78 (51.7)	73 (48.3)	1	**NS**				
Female	62 (60.8)	40 (39.2)	0.69	0.41–1.15				
**Professional Cadre**								
Medical Officer	76 (52.8)	68 (47.2)	1	**φ**		**φ**		**φ**
Nurses	29 (76.3)	9 (23.7)	0.35	0.15–0.78	0.31	0.11–0.83	0.32	0.12–0.85
Clinical Officer	15 (51.7)	14 (48.3)	1.04	0.47–2.32	1.27	0.39–4.09	2.18	0.55–8.71
Physician/Consultant	16 (42.1)	22 (57.9)	1.53	0.75–3.16	1.04	0.35–3.10	0.71	0.24–2.14
**Work Setting**								
Hospital Ward	99 (58.9)	69 (41.1)	1	**§**		**NS**		**NS**
HIV/AIDS Clinic	20 (37.7)	33 (62.3)	2.36	1.25–4.47				
Health Centre	11 (55)	9 (45)	1.17	0.46–2.98				
**Clinical experience (years)**								
<3	71 (67)	35 (33)	1	**§**		**NS**		**NS**
3–6	28 (44.4)	35 (55.6)	2.54	1.34–4.81				
7–9	13 (38.2)	21 (61.8)	3.28	1.47–7.30				
>9	18 (51.4)	17 (48.6)	1.91	0.88–4.17				
**PHAs seen in past year**								
>100	57 (43.2)	75 (56.8)	1	**§**		**φ**		**φ**
50–100	35 (60.3)	23 (39.7)	0.50	0.27–0.94	0.76	0.31–1.88	0.60	0.26–1.38
<50	46 (74.2)	16 (25.8)	0.26	0.14–0.51	0.34	0.14–0.86	0.32	0.14–0.77
**PHAs seen >50 years**								
<25%	70 (51.1)	67 (48.9)	1	**NS**				
>25%	43 (51.8)	40 (48.2)	0.97	0.56–1.68				
**Highest Education**								
Degree	101 (56.4)	78 (43.6)	1	**φ**		***		***
Diploma	20 (71.4)	8 (28.6)	0.52	0.22–1.23	0.21	0.03–1.33	0.25	0.05–1.25
Masters	14 (35)	26 (65)	2.41	1.18–4.91				
**HIV Training**								
Yes	122 (52.8)	109 (47.2)	2.81	1.15–6.83				
No	22 (75.9)	7 (24.1 )	1	**φ**		**NS**		**NS**
**ART Training**								
Yes	58 (54.2)	49 (55.8)	1.11	0.68–1.82				
No	88 (56.8)	67 (53.2)	1	**NS**				
**Co-morbid. Training**								
Yes	26 (41.9)	36 (58.1)	2.08	1.16–3.70	1.39	0.61–3.15	1.91	0.97–3.80
No	120 (60)	80 (40)	1	**φ**		**NS**		***
**Ever had an HIV test**								
Yes	121 (55.1)	105 (44.9)	1.11	0.49–2.52				
No	15 (57.7)	11 (42.3)	1					
**Interest in Geriatrics**								
Yes	130 (57.5)	96 (42.5)	0.58	0.28–1.21				
No	15 (44.1)	19 (55.9)	1	**NS**				

aOR – Odds Ratio adjusted for all variables in the model; CI – Confidence Interval; NS – Not Significant; Level of significance P<0.01 (§), P<0.05 (φ) and P<0.1 (***); Probability>F is <0.001 for all variables in the imputed model.

Compared to medical officers, clinical nurses of various levels of qualification were less knowledgeable (OR = 0.35, 95% CI; 0.15–0.78). More clinicians who had attended to less than 100 and less than 50 PHLWAs respectively in the preceding year scored 50% and below on the knowledge scale compared to those who saw over 100, (OR = 0.50, 95% CI; 0.27–0.94; OR = 0.26, 95% CI; 0.14–0.51, respectively).

However, in the multivariable logistic regression model only clinician age (30–39 years old, aOR = 3.28, 95% CI; 1.65–9.75), PHLWAs seen in the past year (less than 50, aOR = 0.34, 95% CI; 0.14–0.86), and professional cadre level (clinical nurse practitioner; 0.31∶0.11–0.83) were independently associated with knowledge scores about HIV/AIDS in older adults (Table 3). The association between having a diploma qualification and lower knowledge scores was marginal (aOR = 0.21, 95% CI; 0.03–1.33; P = 0.09).

### Missing Data

The following baseline variables were missing arbitrarily : years of clinical experience (24), work setting (21), educational qualification (15), professional cadre (13), PLWHAs attended to in the past year (10), gender (9), age (8) and additional HIV/AIDS training (2) totaling to 65 (22.9%) incomplete cases in the adjusted regression model. We found small to moderate correlations between missingness of a given baseline variable and specific questions with the largest absolute values ranging from 0.16 to 0.22, consistent with our assumption of Missing At Random. The imputed model was not adversely different from the complete cases analysis (Table 3).

### Qualitative Thematic Analysis

Three themes emerged from content analysis namely, a need for clinician training for chronic care, and the associated enablers and barriers.

### A Need for Clinician Training to Care for Older Adults

Respondents had varying views about training clinicians to care for older adults. These were either specific to older adults or general issues of patient care. On the one hand, a respondent acknowledged that curricula offered in the training schools did not pay sufficient attention to the care for older persons:

“*…You go to the training institutions in health, (and you find) their knowledge (and awareness) on (care for older persons) geriatrics is very low. There is no special attention (given to) geriatrics (care for older persons) and as a result the older people suffer more…*” [Policy maker and researcher]

On the other hand, a different respondent recalled her experiences as a medical student and observed that while clinician training had generally evolved, clinician–patient interaction could be improved irrespective of the patients’ age category:

“*…No! Clinician training needs to (change); it has changed a bit…they are doing a bit of psychology now in the medical school which we didn’t do; we are now trying to get them to do a bit of communication skills, communication! 50% of our patients need just talking therapy which is counseling. The clinicians never do it…*” [Clinician and policy maker]

### Opportunities for Clinician Training in HIV/AIDS Care for Older Adults

Respondents identified three prospects for training clinicians to care for older adults. Foremost, one respondent cited the current HIV/AIDS epidemic which has matured and resulted in more chronic users of antiretroviral drugs and in older adults too. Another referred to an existing short course in advanced antiretroviral therapy management provided by one centre of excellence in HIV/AIDS care whilst the third alluded to training efforts by three government agencies in Uganda, as illustrated below:

“*…Then of course, a priority is training of health workers from the emergency mode to a sustainable mode. I think the emergency mode has been mainly to get people (with HIV/AIDS) to treatment; you tell them about adherence, what the drugs are for, and we off-load them to the treatment unit. The sustainable (HIV/AIDS care plan) is now considering the possible complications of the long-term therapy. That by giving consideration to the management of the elderly or aged people on the treatment; how can we manage?*” [Clinician, activist and researcher]

“*…I think it’s important that the people (clinicians) get the training now so that the mind set is already there; once these (aging in HIV/AIDS) reach a certain critical point. The (HIV/AIDS care centre of excellence) offers an advanced ART course within which we talk about some co-morbidities (including non-communicable diseases); we talk about toxicities, Hepatitis B … what might be lacking is consolidating this into a module that looks at aging in particular…*” [Clinician and researcher]

“*…we are now developing a manual and in this manual we are integrating geriatrics with gerontology. We have got support from World Health Organization…but we have worked hard with the (relevant policy body) to ensure that issues of geriatrics and gerontology are included in the training programs. In (a particular university), has a course unit in social gerontology under the department of social work and social administration, and also under the department of community based rehabilitation of people with disabilities?*” [Policy maker and researcher]

### Potential Barriers to Training Clinicians in the Care for Older Adults with HIV/AIDS

Respondents identified various potential barriers to training clinicians to care for older individuals with HIV/AIDS. Some appeared to arise from low awareness and poor attitudes towards care for older persons, while others seemed to be more fundamental beyond a clinicians influence. Responses varied from having little interest in caring for the elderly, to clinicians declining to take courses in geriatrics even when the opportunities were at their disposal. One respondent noted:

“*…I carried out a study there (clinician training institution) about the aging issues; one respondent said…we are more interested in the under – fives; why bother with those, the expired (older adults)… and then another one on HIV and AIDS… they think that older persons are not sexually active and therefore they are not targeted…*” [Policy maker and researcher]

And on interest of healthcare workers in being trained:

“*…In fact one time we wanted to sponsor a nurse to go for a course in Malta in geriatrics but they (health workers in a public hospital) seemed not to be interested. I went to the NGO (non-governmental organization) that’s where I got a nurse from … and she went (for the training)…*” [Policy maker and researcher]

A policy maker observed that while absenteeism from work was an important obstacle to training of health workers, those who were trained did not optimize their skills. In addition, she lamented over staff attrition in search for greener pastures outside the health sector and abroad.

“*…Those who are trained are not there (health care workers absent at facilities); we have no retention strategy, we have no motivation strategy. We have continuously trained apparently but (there is) no recruitment…*” [HIV/AIDS policy maker]

## Discussion

Aging in HIV/AIDS is an emerging area of interest that has received more attention in High- and Middle-Income Countries than in sub-Saharan Africa, including Uganda [13], [31], [32]. In fact, until recently, the Joint UN Programme on HIV/AIDS annual epidemic update did not have data on adults older than 50 years, instead concentrating on the 15–49 years age range [33]. Similarly, Uganda’s 2004/05 national HIV sero-prevalence survey report lacks comprehensive data on persons above 50 years, stating “…*since most of the internationally accepted HIV/AIDS indicators are based on the population aged 15–49*…,” [34]. The HIV prevalence for the 50–54 year olds in Uganda is 6.1% [34]. Regional data from Kenya shows that the prevalence of HIV notification in men aged 50–54 years increased significantly from 5.7% in 2003 to 9.1% in 2008, supporting the need to prioritize this population group [35], [36].

In this study we report areas of sub-optimal clinician knowledge about older adults with HIV/AIDS. We also identified that extremes of age; fewer number of PLWHAs seen; being a clinical nurse or having diploma education level were independently associated with lower knowledge scores about aging and HIV/AIDS.

Although most clinicians accurately stated Uganda’s national HIV sero-prevalence rate of 6.4%, very few pointed out that the burden is higher in 50–59 year older adults than the youth aged 15–24 years [34]. Indeed aging may reduce the perception of risk of acquiring and transmitting HIV and other sexually transmitted infections through unprotected sex, as shown by findings from our qualitative interviews. This “neglect” may be further augmented by the relatively lower life expectancy in the poor countries of sub-Saharan Africa, relegating care for older adults as a health priority. Given that older persons are not considered a target group, they miss out on HIV prevention and care interventions. This also means that, health systems are less likely to respond to the additional burden of care in older HIV-infected persons in terms of financing, infrastructure and human resources training.

Despite a high self-reported interest in care of older individuals, most clinicians scored 50% or below. Questions on HIV/AIDS co-morbidities and long term antiretroviral-therapy toxicities attracted lower scores compared to epidemiology and prevention. The data also suggest that more clinicians with additional HIV/AIDS training had a knowledge score above 50%. However, this was not sustained in the multivariable model indicating that a critical mass is yet to be reached in order to make a difference in terms of transfer of knowledge on care for aging PLWHAs. Only 23% had completed short courses in HIV/AIDS co-morbidities and toxicities following chronic antiretroviral-therapy use, demonstrating that this topic should be given priority in future comprehensive HIV care trainings.

Clinician age independently predicted knowledge scores, consistent with two previous studies among physicians in New York, USA [20] and Britain [37]. More clinicians in their fourth decade of life had knowledge scores above 50% than those aged less than 30 or above 40 years. Older clinicians were probably not consistently exposed to prescribing antiretroviral drugs early in their careers or if they were, then their knowledge has decayed. This finding is augmented by the higher and significantly different unadjusted odds ratios for clinician experience between 3–9 years compared to less than 3 years and above 9 years. These results call for in-service refresher courses for older clinicians, and pre-service training.

Clinical nurses had significantly less knowledge scores compared to general medical doctors. In addition clinicians with a diploma qualification had lower knowledge, with borderline significance in the adjusted analysis. This group typically consisting of clinical nurses and clinical officers, are increasingly taking over duties from medical doctors as HIV clinics become more congested, cohorts become clinically stable, grow older, HIV/AIDS funding becomes less available and to some extent due to brain-drain [38]. Hence, systematic task-shifting should go beyond classroom training and encompass onsite mentorship, coaching, continuous medical education and regular consultation on chronic care for PLWHAs by competent senior clinicians.

The estimated number of PLWHAs seen in the preceding 12 months independently predicted knowledge scores. Those who estimated that they had seen fewer than 50 PHLWAs had less knowledge scores irrespective of work setting. In this line, clinicians who practiced mainly in HIV/AIDS specialist centres did not have higher knowledge scores in the regression model. On the one hand, this finding may imply that quality HIV/AIDS care and knowledge has diffused into the primary health care system in Uganda so that it does not matter where clinicians practice. On the other hand, selection of target groups for trainings should not assume clinicians in HIV/AIDS clinics are more knowledgeable and they should be equally prioritized.

Findings from the qualitative interviews suggest that medical and nursing schools may have lagged behind in pre-service training of clinicians in the care for older adults. These institutions should optimize the mature HIV/AIDS cohorts and budding training opportunities to kick-start short – courses in HIV/AIDS care for older adults in Uganda. In addition, communication skills’ training is vital. Good communication facilitates managing concerns of older adults whilst providing HIV/AIDS prevention and care. For example, during HIV counseling and testing younger clinicians eliciting sexual history from older adults in an African cultural context presents a challenge.

This study was not without limitations. The urban study sample of clinicians may not represent those in rural HIV care centres. However, it is likely that clinicians isolated in rural settings are less knowledgeable, since access to updated medical evidence and opportunities for consultation or training are limited making our study findings even more relevant. Secondly, a human resources for health survey showed that 71% of medical doctors are in the central region where Kampala falls, making our study representative [39]. Although we employed consecutive sampling, clinicians were enrolled from all the 19 major health units of Kampala district which provide care to an estimated over 60% and 90% of the general and HIV/AIDS populations respectively. This is in line with the 20/80 Pareto principle here-in interpreted as 20% of the health facilities handle 80% of the HIV/AIDS burden, and confirmed by reanalysis of the ministry of health database [40], [41], [42]. In addition, this strategy was more feasible and resulted in a high response rate with 182 medical doctors yielding a representative sampling fraction of about 20% in Kampala district. Missing data was explored statistically and did not show significant impact on the overall findings. Not least, some respondents complained of the long exam–like questionnaire with the anticipation of under-reporting their level of knowledge and practice about HIV/AIDS in older adults.

### Conclusions

Our study demonstrated gaps and differences in clinician knowledge that need to be addressed. Clinician HIV/AIDS training and practice has focused on management of acute opportunistic infections, antiretroviral therapy initiation and acute toxicities, and prevention of mother-to-child transmission. This situation is likely to be similar in other low and middle income countries affected by HIV/AIDS. With increased access to antiretroviral drugs and improved patient outcomes, the government health and education departments in sub-Saharan Africa should consider training clinicians to meet the challenges of comprehensive chronic care for older adults with HIV/AIDS.

## Supporting Information

Figure S1 **Survey Questionnaire for the Aging & HIV/AIDS in Older Adults Study.**
(DOC)
