# Supplementary material for: Determinants of Clinician Knowledge on Aging and HIV/AIDS: A Survey of Practitioners and Policy Makers in Kampala District, Uganda
Source: PLoS One. 2013 Feb 28;8(2):e57028. doi: 10.1371/journal.pone.0057028 (PMC3585272; doi:10.1371/journal.pone.0057028)
Supplement: Figure S1 — Survey Questionnaire for the Aging & HIV/AIDS in Older Adults Study. (DOC) [file pone.0057028.s001.doc]

**Figure S1: Survey Questionnaire for the Aging & HIV/AIDS in Older Adults Study**

**SURVEY QUESTIONNAIRE FOR THE AGING & HIV/AIDS IN OLDER ADULTS STUDY**

**(AgHOA)**

1. Unique Identification Number:

|  |  |  |  |
| --- | --- | --- | --- |

1. Initials of Interviewer:*(Three Capital Letters)*

|  |  |  |
| --- | --- | --- |

1. Date:

| Day | Month | Year |
| --- | --- | --- |
|  |  | 2011 |

1. Time: *(Tick One)*

| 07:00 – 11:59 HRS | 12:00 – 16:59 HRS | 17:00 – 19:00 HRS |
| --- | --- | --- |
|  |  |  |

**Part A - Please complete the following questions:**

**1**. How old are you? ____________________

**2**. What is your gender? ⬜ Male ⬜ Female

**3**. What is your professional cadre level?

⬜ Clinical Officer ⬜ Medical Officer ⬜ Specialist ⬜ Consultant ⬜ Clinical Nurse

⬜ Other (specify) ________________________

**4**. What is the setting of your work?

⬜ Health Centre (Level) ______________ ⬜ Hospital (department/ward) _________________

⬜ HIV/AIDS clinic ⬜ Area of interest (e.g. TB/HIV) ________________________________

**5**. How many years of clinical working experience do you have? _____________________________________________________

**6**. What is your highest level of clinical education?

⬜ Diploma ⬜ Higher Diploma ⬜ Degree ⬜ Masters ⬜ PhD

⬜ Specific qualification (e.g. MMED or MSc etc) ________________________

**7**. Have you received any additional training in HIV/AIDS?

⬜ No ⬜ Yes ⬜ VCT/RCT ⬜ TB/HIV ⬜ PMTCT ⬜ SRH/STI ⬜ Other OIs

⬜ Conferences ⬜ Co-morbidities ⬜ Paed. & Adol. ⬜ ART

**8**. Are you interested in HIV/AIDS interventions for older adults (Geriatrics)?

⬜ Yes ⬜ No

**9**. Have you been tested for HIV before?

⬜ Yes ⬜ No

**Part B - HIV/AIDS Knowledge Scale: *Please answer the following questions as: T-True; F-False or DK-Don’t Know. Please do not guess if you do not know. Thank you.***

**1**. The prevalence of HIV/AIDS in Uganda is 6.4%? ⬜ T ⬜ F ⬜ DK

**2**. In Uganda, the prevalence of HIV/AIDS among older adults (50-59 yrs) is higher than younger adults (15-24 yrs)?

⬜ T ⬜ F ⬜ DK

**3**. Among Ugandan older adults HIV/AIDS is predominantly transmitted by: a) Casual contact ⬜ T ⬜ F ⬜ DK b) Blood Transfusion ⬜ T ⬜ F ⬜ DK c) Sweat, saliva and tears ⬜ T ⬜ F ⬜ DK d) Homo and Hetero-sexual intercourse ⬜ T ⬜ F ⬜ DK

**4**. Gloves are **not** necessary when handling body fluids from older adults? ⬜ T ⬜ F ⬜ DK

**5**. Hetero-sexual intercourse is **not** the most important risk factor for transmission of HIV/AIDS among older adults?

⬜ T ⬜ F ⬜ DK

**6**. An older adult without HIV/AIDS symptoms can be infectious? ⬜ T ⬜ F ⬜ DK

**7**. An older adult may be infected with the HIV/AIDS germ even if he/she tests negative for HIV antibodies?

⬜ T ⬜ F ⬜ DK

**8**. HIV/AIDS is characterized by a decrease in CD4+ T-lymphocyte cells in the blood? ⬜ T ⬜ F ⬜ DK

**9**. An older adult with antibodies to the HIV virus is protected against HIV/AIDS? ⬜ T ⬜ F ⬜ DK

**10**. HIV/AIDS is characterized by chronic immune activation **but not** chronic inflammation? ⬜ T ⬜ F ⬜ DK

**11**. Is chronic **immune activation** in the context of HIV associated with any of the following? a) Aging ⬜ T ⬜ F ⬜ DK b) Atherosclerosis ⬜ T ⬜ F ⬜ DK c) Malignancy ⬜ T ⬜ F ⬜ DK d) Metabolic Syndrome ⬜ T ⬜ F ⬜ DK e) Osteoporosis ⬜ T ⬜ F ⬜ DK f) Immune senescence ⬜ T ⬜ F ⬜ DK

**12**. Is chronic **inflammation** in the context of HIV associated with any of the following? a) Aging ⬜ T ⬜ F ⬜ DK b) Atherosclerosis ⬜ T ⬜ F ⬜ DK c) Malignancy ⬜ T ⬜ F ⬜ DK d) Metabolic Syndrome ⬜ T ⬜ F ⬜ DK e) Osteoporosis ⬜ T ⬜ F ⬜ DK f) Immune senescence ⬜ T ⬜ F ⬜ DK

**13**. Myocardial Infarction (heart attack) occurring in an older adult with HIV/AIDS is purely regarded as an event of the immune system: ⬜ T ⬜ F ⬜ DK

**14**. HIV/AIDS causes earlier ageing of the body and is specifically associated with different prematurely occurring clinical outcomes:

⬜ T ⬜ F ⬜ DK

**15**. HIV positive older adults have the same risk of co-morbidities (diabetes mellitus, non-AIDS related cancers, cardiovascular disease etc.) as HIV negative older adults: ⬜ T ⬜ F ⬜ DK

**16**. All pregnant women infected with HIV will have babies born with AIDS: ⬜ T ⬜ F ⬜ DK

**17**. Antiretroviral therapy can reduce the risk of mother to child transmission to nearly 0%: ⬜ T ⬜ F ⬜ DK

**18**. Effective ART isn’t a cure but can improve average life expectancy of HIV infected older adults to as good as that of the HIV negative general population: ⬜ T ⬜ F ⬜ DK

**19**. The life expectancy of PHAs taking effective ART is similar to the general population only if CD4+ counts are ≥500 cells/mm^3^ for ≥5 years:

⬜ T ⬜ F ⬜ DK

**20**. Even after 5 years of effective ARVs, many patients fail to obtain normal CD4+ T-cell counts:

⬜ T ⬜ F ⬜ DK

**21**. Older adult PHAs with CD4+ <500 cells/mm^3^ after about 5 years of ARV use are unlikely to achieve ≥500 cells/mm^3^ during long-term follow-up:

⬜ T ⬜ F ⬜ DK

**22.** Older adults with HIV respond less well to ARVs compared to younger adults: ⬜ T ⬜ F ⬜ DK

**23.** The following is the correct order of factors increasing the risk for suffering TB disease: a) DM>Smoking>HIV/AIDS ⬜ T ⬜ F ⬜ DK b) Smoking>DM>HIV/AIDS ⬜ T ⬜ F ⬜ DK c) HIV/AIDS>DM>Smoking ⬜ T ⬜ F ⬜ DK d) DM >HIV/AIDS>Smoking ⬜ T ⬜ F ⬜ DK e) Smoking> HIV/AIDS>DM ⬜ T ⬜ F ⬜ DK f) HIV/AIDS>Smoking>DM ⬜ T ⬜ F ⬜ DK

**24.** Which of the following are examples of Serious Non-AIDS Related Events (SNARES) which occur more frequently in older adults with HIV/AIDS even in the presence of effective ART use than in HIV negative older adults: a) Renal disease ⬜ T ⬜ F ⬜ DK b) Hepatoma ⬜ T ⬜ F ⬜ DK c) Cardiovascular disease ⬜ T ⬜ F ⬜ DK d) Anal cancer ⬜ T ⬜ F ⬜ DK e) Non-Hodgkin’s Lymphoma ⬜ T ⬜ F ⬜ DK

**25**. Older patients, at the time of initial HIV diagnosis, tend to have higher CD4 cells than younger individuals:

⬜ T ⬜ F ⬜ DK

**26**. The CD4+ cells peak response and plateau of older adults on the same ART regimen is lower than that of younger individuals:

⬜ T ⬜ F ⬜ DK

**27.** Older adults are likely to have undetectable viral loads more than younger individuals: ⬜ T ⬜ F ⬜ DK

**28.** Older patients with HIV have a shorter survival after a diagnosis of AIDS compared to younger patients:

⬜ T ⬜ F ⬜ DK

**29.** Older adults are less likely to use condoms, more likely to have multiple partners and those who are HIV infected adhere less to ART than younger individuals: ⬜ T ⬜ F ⬜ DK

**30.** Untreated HIV infection in older adults leads to protein energy malnutrition: ⬜ T ⬜ F ⬜ DK

**31.** The following conditions constitute AIDS wasting syndrome; 10% loss of weight plus: a) Chronic fever ⬜ T ⬜ F ⬜ DK b) Cryptococcal meningitis ⬜ T ⬜ F ⬜ DK c) Chronic diarrhea ⬜ T ⬜ F ⬜ DK d) Chronic weakness ⬜ T ⬜ F ⬜ DK e) Cryptosporidiosis ⬜ T ⬜ F ⬜ DK **32.** ART causes **initial** loss of both central and limb fat (lipoatrophy) in older adults: ⬜ T ⬜ F ⬜ DK

**33.** After a period of time, ART causes gain of limb fat and central abdominal fat in older adults: ⬜ T ⬜ F ⬜ DK

**34.** Long term use of ART may cause redistribution of body fat increasing risk of Diabetes Mellitus in older adults:

⬜ T ⬜ F ⬜ DK

*********************************************************************************

**Thank You. I would like to receive a copy of the survey results.**

⬜ No ⬜ Yes **Cell number (only if yes): _____________**____________

*****************END****************END**************END******************
